# Surface Roughness Analysis of H13 Steel during Electrical Discharge Machining Process Using Cu–TiC Sintered Electrode

**DOI:** 10.3390/ma14205943

**Published:** 2021-10-10

**Authors:** Arminder Singh Walia, Vineet Srivastava, Mayank Garg, Nalin Somani, Nitin Kumar Gupta, Chander Prakash, Cherry Bhargava, Ketan Kotecha

**Affiliations:** 1 Department of Mechanical Engineering, Thapar Polytechnic College, Patiala 147004, India; arminderwalia@gmail.com; 2 Department of Mechanical Engineering, Thapar Institute of Engineering and Technology, Patiala 147004, India; Vineet.srivastava@thapar.edu (V.S.); mayank.garg@gmail.com (M.G.); 3 Department of Mechanical Engineering, DIT University, Dehradun 248009, India; 4 Department of Mechanical Engineering, ABES Engineering College, Ghaziabad 201001, India; ghotnitin@gmail.com; 5 School of Mechanical Engineering, Lovely Professional University, Phagwara 144402, India; chander.mechengg@gmail.com; 6 Department of Electronics and Telecommunication Engineering, Symbiosis Institute of Technology, Pune 412115, India; cherry.bhargava@sitpune.edu.in; 7 Symbiosis Centre for Applied Artificial Intelligence (SCAAI), Symbiosis International (Deemed University) (SIU), Pune 412115, India

**Keywords:** EDM, cermet tooltip, surface roughness, dimensional analysis, pulse current, pulse duration, pulse interval

## Abstract

In electrical discharge machining (EDM), the machined surface quality can be affected by the excessive temperature generation during the machining process. To achieve a longer life of the finished part, the machined surface quality plays a key role in maintaining its overall integrity. Surface roughness is an important quality evaluation of a material’s surface that has considerable influence on mechanical performance of the material. Herein, a sintered cermet tooltip with 75% copper and 25% titanium carbide was used as tool electrode for processing H13 steel. The experiments have been performed to investigate the effects of EDM parameters on the machined surface roughness. The findings show that, as the pulse current, pulse length, and pulse interval are increased, the surface roughness tends to rise. The most significant determinant for surface roughness was found to be pulse current. A semi-empirical surface roughness model was created using the characteristics of the EDM technique. Buckingham’s theorem was used to develop a semi-empirical surface roughness prediction model. The semi-empirical model’s predictions were in good agreement with the experimental studies, and the built empirical model based on physical features of the cermet tooltip was tested using dimensional analysis.

## 1. Introduction

The electrical discharge machining (EDM) machine tool has been effectively and efficiently used to cut/process high-strength materials. In EDM, the repetition of electrical sparks between electrodes is used for melting and helps remove the material from the workpiece. During the operation, in the machining zone, the temperature can reach up to 8000–10,000 °C [1], which results in the heating, melting, as well as evaporation of work material and the electrode material. As the supply is withdrawn, the developed plasma channel is implored by the circulating dielectric. During the operation, as soon as the supply of input current stops, a pressure or shockwave will be created. This shockwave will break the plasma channel and a crater will be formed at the site. The repeated electrical discharges of very high temperature leads to the flushing of molten material from the machining site as microscopic chips [2,3,4,5]. The EDM process has many advantages, including its ability to machine a large range of electrically conductive materials regardless of their hardness, its high accuracy, the lack of requirement for any post machining in most cases, etc. However, the EDM process is not free of problems, such as electrode wear, low material removal during the machining of hard materials and hardened steels, and change in the profile of the tool during machining, especially the materials with high hardness values [1,2]. Due to its higher conductivity, Copper is widely used as a tool material, and this tool can be fabricated by different techniques [5,6,7]. However, it has been observed that the Cu tool wears out while machining harder materials [8]. As the EDM process is a replica process [9], any variation in tool shape due to disintegration will have a direct impact on the work material roughness. Various modifications have been suggested by researchers to avoid tool wear. The EDM process is controlled by process variables such as pulse current, pulse duration, pulse interval, gap voltage, dielectric flushing pressure, duty factor, polarity, etc., which directly influence the process performance. These days, artificial intelligence (AI) contributes significantly to all fields of engineering. It plays an important role in every industry, especially in the machining industry. The technologies and techniques equipped with AI applications are widely used in machining processes. It has been found that several approaches, such as thermal modelling, artificial neural network (ANN) [10,11,12,13], dimensional analysis [14], firefly algorithm [15], grey relational technique (GRA) [16], response surface methodology [17,18], and the Taguchi [19] and finite element methods (FEM) [20], have been adopted to model and simulate surface roughness (SR) and tool shape.

A semi-empirical model to predict surface roughness with the help of the dimensional analysis method during EDM has been reported [21,22,23]. The model was successful in predicting the response with an error of up to 12%. Payal et al. [13] used a regression model in order to estimate the MRR and TWR. The sufficiency of the model was justified by ANOVA. The developed model was successful to predict MRR and TWR with an accuracy of 94.65 and 96.91%, respectively. Patil and Brahmankar [23] determined the semi-empirical model of MRR during WEDM of a metal matrix. The model was successful in predicting the MRR with more than 99% accuracy. The ANN was used to predict the SR [24] in the EDM process. Both the models of MATLAB and Netlab were successful in providing reliable predictions. Bobbili et al. [25] used dimensional analysis effectively to investigate the process parameters on SR and MRR of homogeneous rolled armor and aluminum alloy 7017. Talla et al. [26] applied the regression analysis and hybrid dimensional approach for evaluating the output in terms of SR and MRR. Guo et al. [27] optimized machining variables for the EDM process using the Taguchi and grey relational analyses. Mohanty et al. [28] applied the utility concept and particle swarm optimization (PSO) and studied the tool wear rate. QSPO obtained favorable results with a higher utility index (6.21) than PSO (5.89). ANN was used by Janmanee and Kumjing [29] to study the microcracks during the EDM of WC-Co based composite. Walia et al. [30] utilized the tool material’s physical and electrical properties and developed a semi-empirical based model to calculate the changes in tool shape for EDM. The prediction error for the validation experiment varied from 3.38–4.62%. Perez [16] used a fuzzy interference system (FIS) and developed a technological table. The aim of study is to decide the optimum process parameters to maximize or minimize the response according to the requirement.

The performance of the harmony search algorithm was compared with Taguchi grey relational analysis by Mahalingam et al. [17]. The result obtained with HAS proved to be better and the prediction error was less than 6%. Belloufi et al. [31] predicted the performance of EDM process using fuzzy model during the machining of AISI 1095 treated steel. The model predicted MRR, TWR, SR and radial overcut with an average error of 1.51, 3.386, 5.285, and 4.004%, respectively.

From the above literature, it can be seen that many techniques have been utilized to predict the various responses analyzed in the literature. However, very few models have been formulated to predict the surface roughness during EDM of tool steel utilizing cermet tooltip (Cu–TiC). H13 steel has been used as the work material as it is abundantly used in the manufacturing of dyes, which is the major application area of EDM. The hardened H13 steel was used as the work material because it produces more challenges for the tool to maintain its shape during machining. A round shape Cu–TiC tool has been used. Hence, the current work aims to fill this gap and create a semi-empirical model to predict the surface roughness (SR) during the EDM of H13 steel with a copper–titanium carbide tool electrode. To develop the model, pulse current (I_p_), pulse duration (T_on_), and pulse interval (T_off_) have been utilized as process variables, and physical properties such as linear expansion coefficient, thermal conductivity, electrical hardness, and the density of a material were studied.

## 2. Materials and Method

### 2.1. Materials Synthesis

**Figure 1:**
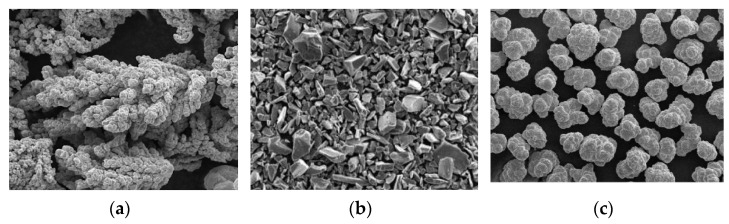
SEM images of (**a**) Cu powder, (**b**) TiC powder, and (**c**) Ni powder.

Cu and TiC powders with grain size of 50 µm (average) were utilized during the study. Figure 1 represents the SEM image of the Cu and TiC and it is evident that the Cu powder particles have dendritic shape, while TiC particles have irregular sharp edges. Similar findings have been observed by walia et al. [8,22].

### 2.2. Preparation of the Tool

In this work, a copper–titanium carbide (Cu–TiC) ceramic tool was prepared for the EDM machining process. A tool diameter of 8 mm was decided for machining. The material composition for this ceramic tool was chosen based on the available literature along with the trial experiments, i.e., 75% is of copper powder and 25% is of titanium carbide powder, with an average particle size of 45 µm. A constant percentage of Nickel powder of same particle size and 3% by weight was used to obtain a better densification.

**Figure 2:**
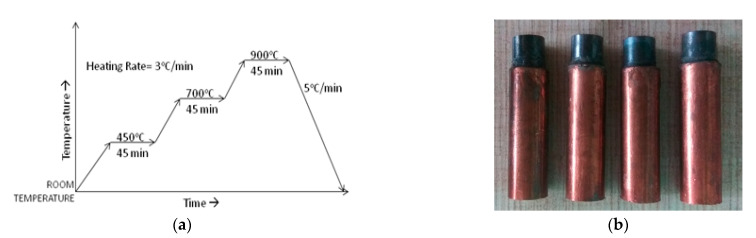
(**a**) Sintering cycle used in this study; (**b**) brazed Cu–TiC tools.

Copper powder and titanium carbide powder were mixed with the help of the ball mill machine at 25 rpm for 40 min to obtain a homogeneous mixture. A tungsten carbide (WC) die has been fabricated to create a pallet of the ceramic. The powder mixture was filled in the die, and compacting was done at the pressure of around 10 kg/mm^2^ for 15 min holding time for each sample. Then, these samples were sintered in a tubular furnace (Victory Sensors, HTF-006). The temperature of the furnace can be controlled to an accuracy of ±2 °C. Before the sintering process, the alumina tube was flushed with argon gas. The sintering process was conducted in three stages, i.e., 450, 700, and 900 °C, with 45 min holding time using a heating schedule of 3 °C/min. Figure 2a represents the sintering cycle. Then, these sintered pallets were brazed to a copper rod to make it convenient for tool holding in an EDM machine. The final Cu–TiC ceramic tool used for the machining process is shown in Figure 2b.

### 2.3. Workpiece Preparation

**Figure 3:**
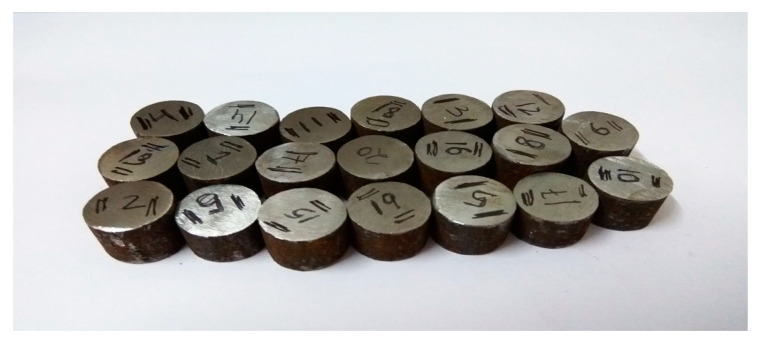
H13 work pieces.

H13 steel (50 mm × 50 mm × 15 mm) was taken as the workpiece in this work. The surface of the workpiece was initially finished on a disk polishing machine (Radical Instruments, Panchkula, India Model: RPM-33D) to obtain a uniform machining surface as shown in Figure 3. Then, energy dispersive X-ray spectroscopy (EDS) (JEOL, Peabody, MA, USA, Model-JSM 6610 LV) was performed on a non-machined workpiece to determine its composition. The percentage of composition is presented in Table 1.

### 2.4. Properties Measurement of Tool Material

The surface roughness produced with a tool material is influenced by the hardness [1]. A harder tool will have a more abrasive action on the machined surface. Therefore, while performing this study, the hardness of the materials was considered an important factor. In addition, the electrical and thermal conductivity of the electrode has a crucial role in the machining, as the heat generated in the zone of tool workpiece interface impacts the surface roughness. The amount and particle size of material removed in a single spark depends on the number of ions and electrons that make a strike with the work surface, which is directly dependent upon the electrical conductivity of the tool material. Hence, the most significant properties of the tool material (electrical and thermal conductivity, hardness, and material density) impacting the surface roughness of the machined cavity were measured using different techniques.

The four-point probe method (DMV-001, Roorkee) and Lorenz law [32] were used to calculate the electrical and thermal conductivity, respectively. The dispersion range for the electrodes regarding electrical conductivity was found to be from −2.33 to +1.69%. Thermal conductivity varies linearly with electrical conductivity [2,3,7,30]. Therefore, Lorenz law [32] was applied to evaluate the thermal conductivity of the materials. The micro-hardness of the samples was assessed by a Vickers micro-hardness tester (Struers, Westlake, OH, USA, Model-MVH-1) at an indentation load of 0.5 kgf and 20 s dwell time. Each measurement was repeated five times for every sample. Table 2 represents the average measured values of the properties.

### 2.5. Selection of Process Parameters

The EDMing performance on tool steel is dependent on several dependent variables [33]. These variables can be categorized as electrode-based parameters, workpiece material-based parameters, electrical, and non-electrical variables. The literature states that the I_p_, T_on_, and T_off_ are the most significant parameters that influence the EDM process. Therefore, these three factors have been chosen for the current research. The EDM has been done by considering these three factors at various levels. Table 3 summarizes the ranges of values for these three factors, which were decided according to the literature survey and machine capabilities.

### 2.6. Experimentation on EDM with Cu–TiC Tooltip

Die sinking EDM (ELECTRONICA VCP 20) was used for experimenting with the Cu–TiC ceramic tool. In every experiment, the dielectric medium was kerosene oil. A total of 20 experiments were carried out using CCRD-based optimization (Design expert) with independent factors at 5 different levels. The machining time was kept as 15 min for each workpiece. An SR tester (Mitutoyo, Aurora, IL, USA, SJ-400) was used during the experimentation in order to measure the response of average SR (R_a_), which can evaluate the shape of micro relief [34]. Additionally, 0.8 mm was set as the cut-off length, while the sampling length was set as 5 mm. The measurement was repeated five times for every sample. The average value of the five readings was considered the response. Table 4 shows the measured readings of surface roughness for the conducted experiments.

## 3. Modelling of Surface Roughness Using Dimensional Analysis

In order to generate the empirical model of surface roughness, a dimensional analysis method was used. Using this method, we can deduce the information regarding a phenomenon from the individual premise [35]. Buckingham’s π theorem has been utilized for developing a predictive semi-empirical model of surface roughness. In this model, the physical and thermal properties of machined materials, namely density, thermal conductivity, linear coefficient of expansion, electrical resistivity, and hardness, were considered. The machining process variables, namely I_p_, T_on_, and T_off_, were also considered with material properties.

### 3.1. Buckingham’s π Theorem

It is stated that any equation that relates to dimensionless products is dimensionally homogeneous [36]. Accordingly, if there is an equation with a specific number, ‘n’, of the physical factors, and ‘r’ terms are used to express them, then a set of dimensionless terms p = n − r can be created from the original factors. This gives a technique to discover sets of dimensionless variables from the given factors [36]. This concept is mainly used to collect all factors present in the problem in various dimensionless terms denoted by ‘πs’. Therefore,

(1) $$f\left(\pi_{1},{\;\pi }_{2},\pi_{3},\cdots \cdots \cdots \pi_{n-k}\right)=0$$

### 3.2. Dimensional Analysis for Surface Roughness

Surface roughness is dependent on pulse duration (T_on_), pulse current (I_p_), pulse interval (T_off_), electrical resistivity (R), thermal conductivity (κ), the density of the material (ρ), hardness (HV), and linear thermal expansion (α). Therefore,

(2) $$\mathrm{SR}=f\left(I_{p},T_{\mathrm{on}},T_{\mathrm{off}},R,\;\kappa ,\;\rho ,\;\mathrm{HV},\alpha \right)$$

Table 5 and Table 6 represents the physical properties, units given in Equation (2) and parameter dimensions.

As per the Buckingham π theorem, the desired formula for dimensional analysis formula can be written as (for Equation (2))

(3) $${\left[L\right]}^{K1}{\left[T^{-1}Q\right]}^{K2}{\left[T\right]}^{K3}{\left[T\right]}^{K4}{\left[{\mathrm{ML}}^{3}T^{-1}Q^{-2}\right]}^{K5}{\left[{\mathrm{MLT}}^{-3}\theta^{-1}\right]}^{K6}{\left[{\mathrm{ML}}^{-3}\right]}^{K7}{\left[{\mathrm{ML}}^{-1}T^{-2}\right]}^{K8}{\left[\theta^{-1}\right]}^{K9}=\left[M^{0}L^{0}T^{0}\theta^{0}Q^{0}\right]\;$$

Last 5 columns were used to formulate the square matrix, which was used to find the matrix rank.

$$\left|\begin{array}{cc} \begin{array}{ccc} 1\;\; & \;1 & \;\;\;\;1 \end{array} & \begin{array}{cc} 1 & \;\;0 \end{array}\\ \begin{array}{ccc} 3 & 1 & -3\\ -1 & -3 & 0\\ \begin{array}{c} 0\\ -2 \end{array} & \begin{array}{c} -1\\ 0 \end{array} & \begin{array}{c} 0\\ 0 \end{array} \end{array} & \begin{array}{c} \begin{array}{cc} -1 & \;\;\;\;0 \end{array}\;\\ \begin{array}{cc} -2 & \;\;\;0 \end{array}\\ \begin{array}{cc} \begin{array}{c} 0\\ 0 \end{array} & \;\;\;\begin{array}{c} -1\\ \;0 \end{array} \end{array} \end{array} \end{array}\right|=+4$$

As this is a non-zero 5th determinant, in the current case, there are nine variables and the rank of the matrix is 5, and so there are four dimensionless products.

### 3.3. Dimensionless Products

Depending on the linear algebraic equation, as per Table 5, it can be written as

(4) K5+K6+K7+K8=0

(5) K1+3K5+K6−3K7−K8=0

(6) −K2+K3+K4−K5−3K6−2K8=0

(7) −K6−K9=0

(8) K2−2K5=0 

Solving Equations (4)–(8) for K5, K6, K7, K8, K9, we obtain

(9) $$K5=\frac{1}{2}K2\;;K6=-K1-\frac{3}{2}K2-K3-K4;K7=-\frac{1}{2}K1-\frac{1}{2}K2-K3-K4\;K8=\frac{3}{2}K1+\frac{3}{2}K2+2K3+2K4;K9=K1+\frac{3}{2}K2+K3+K4$$

Assigning the values K1=1;K2=K3=K4=0 in Equation (9), the first solution is K5=0;K6=−1;K7=−1/2;K8=3/2;K9=1

Assigning the values K2=1;K1=K3=K4=0 in Equation (9), the second solution is K5=1/2;K6=−3/2;K7=−1/2;K8=3/2;K9=3/2

Assigning the values K3=1;K1=K2=K4=0 in Equation (9), the third solution is K5=0;K6=−1;K7=−1;K8=2;K9=1

Assigning the values K4=1;K1=K2=K3=0 in Equation (9), the fourth solution is K5=0;K6=−1;K7=−1;K8=2;K9=1

The solutions, in matrix form, are shown in Table 7.

The solution includes an *n–r* row, where

*n (no. of variables)* = 9

*r (matrix rank) =* 5

Hence, in the current case, the below mentioned dimensionless products are found:

$$\pi_{1}=\frac{\mathrm{SR}\times {\mathrm{HV}}^{3/2}\times \alpha }{\kappa \times \rho^{1/2}};\;\pi_{2}=\frac{I_{p}\times R^{1/2}\times {\mathrm{HV}}^{3/2}\times \alpha^{3/2}}{\kappa^{3/2}\times \rho^{1/2}};\;\pi_{3}=\frac{T_{\mathrm{on}}\times {\mathrm{HV}}^{2}\times \alpha }{\kappa \times \rho };\;\pi_{4}=\frac{T_{\mathrm{off}}\times {\mathrm{HV}}^{2}\times \alpha }{\kappa \times \rho }$$

Dimensionless products’ relation can be written as

$$f\left(\pi_{1},{\;\pi }_{2},{\;\pi }_{3},{\;\pi }_{4}\right)=0$$

or

$$\pi_{1}=f\left(\pi_{2},{\;\pi }_{3},{\;\pi }_{4}\right)$$

$$\frac{\mathrm{SR}\times {\mathrm{HV}}^{3/2}\times \alpha }{\kappa \times \rho^{1/2}}=Z\times {\left(\frac{I_{p}\times R^{1/2}\times {\mathrm{HV}}^{3/2}\times \alpha^{3/2}}{\kappa^{3/2}\times \rho^{1/2}}\right)}^{A}\times {\left(\frac{T_{\mathrm{on}}\times {\mathrm{HV}}^{2}\times \alpha }{\kappa \times \rho }\right)}^{B}\times {\left(\frac{T_{\mathrm{off}}\times {\mathrm{HV}}^{2}\times \alpha }{\kappa \times \rho }\right)}^{C}$$

(10) $$\mathrm{SR}=Z\times \left(\frac{\kappa \times \rho^{1/2}}{{\mathrm{HV}}^{3/2}\times \alpha }\right)\times {\left(\frac{I_{p}\times R^{1/2}\times {\mathrm{HV}}^{3/2}\times \alpha^{3/2}}{\kappa^{3/2}\times \rho^{1/2}}\right)}^{A}\times {\left(\frac{T_{\mathrm{on}}\times {\mathrm{HV}}^{2}\times \alpha }{\kappa \times \rho }\right)}^{B}\times {\left(\frac{T_{\mathrm{off}}\times {\mathrm{HV}}^{2}\times \alpha }{\kappa \times \rho }\right)}^{C}$$

The Z (dimensionless constant) and the exponents A, B, and C were obtained by nonlinear approximation of the data. The values of Z, A, B, and C were 8.6806 × 10^−7^, 0.3670, 0.05484, and 0.03645, respectively. As a result, Equation (10) is further simplified and written as

(11) $$\mathrm{SR}=8.6806\times 10^{-7}\times \left(\frac{\kappa \times \rho^{1/2}}{{\mathrm{HV}}^{3/2}\times \alpha }\right)\times {\left(\frac{I_{p}\times R^{1/2}\times {\mathrm{HV}}^{3/2}\times \alpha^{3/2}}{\kappa^{3/2}\times \rho^{1/2}}\right)}^{0.3670}\times {\left(\frac{T_{\mathrm{on}}\times {\mathrm{HV}}^{2}\times \alpha }{\kappa \times \rho }\right)}^{0.05484}\times {\left(\frac{T_{\mathrm{off}}\times {\mathrm{HV}}^{2}\times \alpha }{\kappa \times \rho }\right)}^{0.03645}$$

## 4. Results and Discussion

### 4.1. Experimental v/s Theoretical Surface Roughness

**Figure 4:**
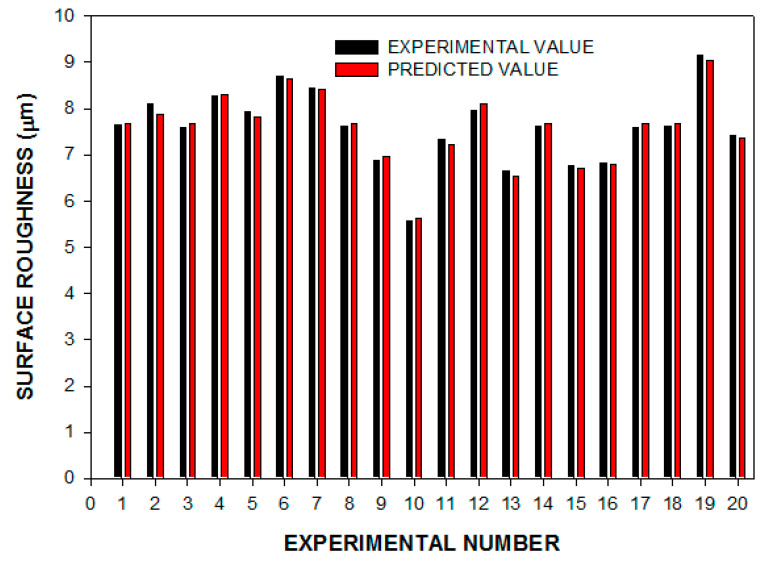
Experimental vs. predicted surface roughness.

The simplified Equation (11) was applied to calculate surface roughness during EDM with sintered cermet tooltip for the machining of H13 steel. It is evident from Figure 4 that the observed and the predicted values have good agreement for the surface roughness during EDM with sintered cermet tooltip. The maximum deviation between the experimental values and the predicted values of the surface roughness were in the range of −1.89% (Experiment 12) to +2.84% (Experiment 2). The developed model was confirmed by experimenting with different machining conditions that were not used to determine Equation (11). Table 8 also represents that the SR can be predicted by the developed model with good accuracy.

### 4.2. Effect of Process Parameters on Surface Roughness

#### 4.2.1. Influence of Pulse Current Variation

**Figure 5:**
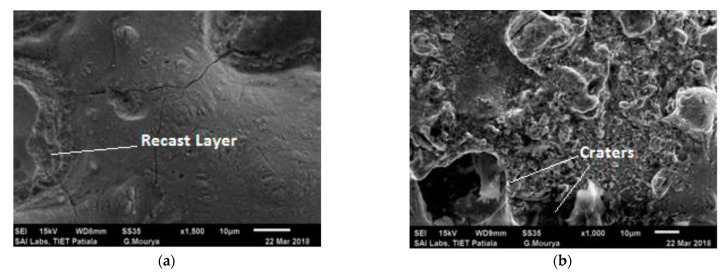
Surface characteristics of a workpiece after EDM (**a**) for Experiment 10 and (**b**) for Experiment 19.

**Figure 6:**
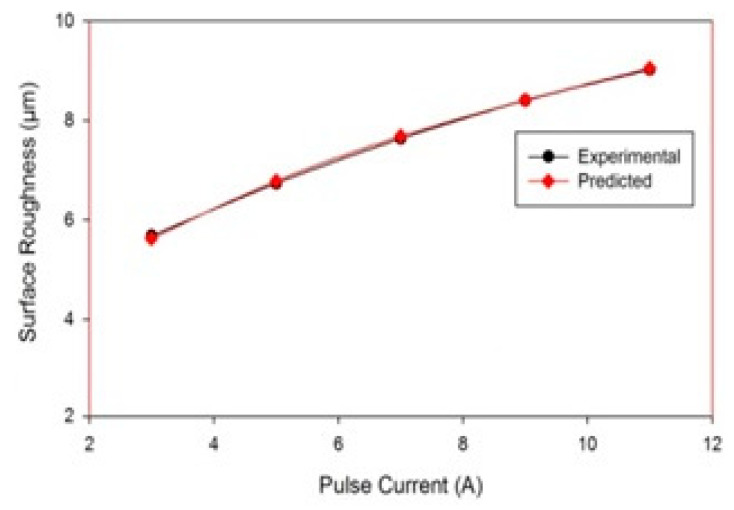
Effect of I_p_ on SR.

The SEM micrographs of the machined work piece by EDM process using Cu–TiC as a tool for low and high value of discharge current at a pulse-on time of 300 μs and pulse-off time of 30 μs are represented in Figure 5. It has been seen from the micrograph that the surface of the machined workpiece is described by an irregular melded structure, shallow holes, globules of debris, and micro-pores. Micrographs also demonstrate the arrangement of a recast layer on the surface of the machined work piece. The development of surface splits can be ascribed to the differentials of compression stress inside the white layer, and when it exceeds the value of ultimate tensile stress of that particular material, this leads to crack formation [8]. It can also be observed from the micrographs that the surface inconsistency increases with an increment in discharge current for both the tool machining. This increment could be due to the formation of deeper and bigger release craters, which results in more damage of surface with the increase in discharge current [15]. Figure 6 represents the outcome of pulse interval on SR. Again, an increase in SR was observed with an increment in the pulse current. Similar results have been observed in the previous studies [17,37,38].

#### 4.2.2. Influence of Pulse on Time Variation

**Figure 7:**
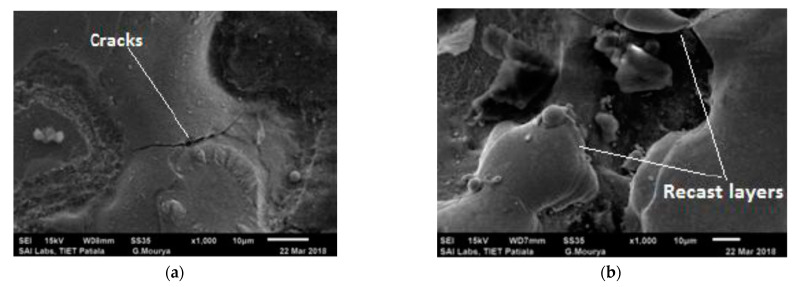
Surface characteristics of the workpiece after EDM (**a**) for Experiment 11 (**b**) for Experiment 2.

**Figure 8:**
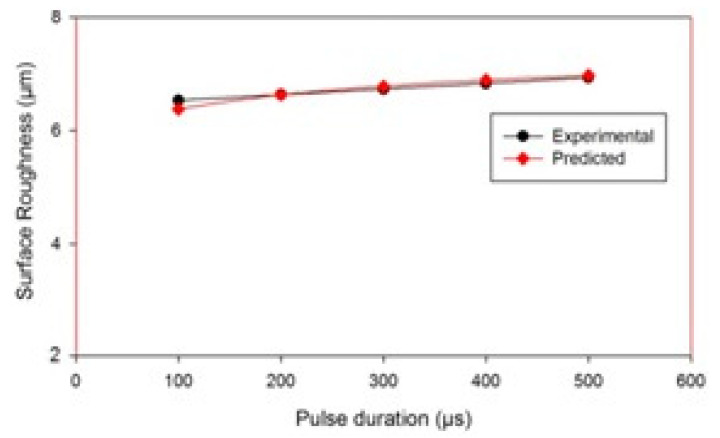
Variation in Surface Roughness with pulse duration.

SEM micrographs of the surface of the machined workpiece by EDM process, using Cu–TiC as the tool material, have been taken for a low and high value of pulse-on time at a discharge current of 7 A and pulse-off time of 30 μs, as represented in Figure 7. It has been observed from the given figures that the increment in surface inconsistencies takes place with the increment in pulse-on time. This might be due to the plasma channel expansion with the increase in pulse-on time [39,40,41], which has augmented the machined zone of workpiece and therefore decreased both the impulsive force and energy density. Due to this, the melting part of debris is not expelled totally and frames an obvious globule-like recast layer to debilitate the surface. These impacts have been seen to be more articulated as the pulse-on time increases. Apart from these irregularities, the micro pores and fine pit developed with the increase in SR. The variation of SR with pulse-on time for Cu–TiC tool can also be observed Figure 8. It can be seen that the SR increases with the increase in the pulse-on time, and the experimental and predicted surface roughness have good agreement.

#### 4.2.3. Effect of Variation in Pulse Interval

**Figure 9:**
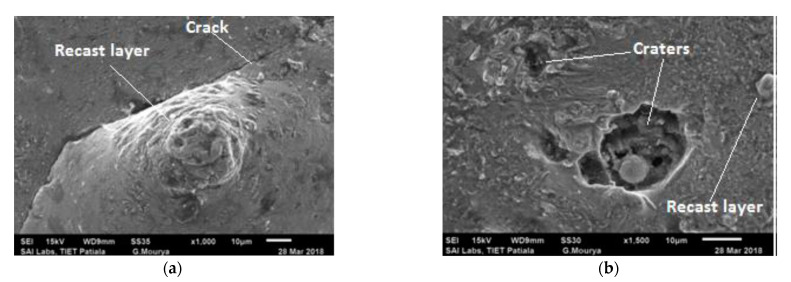
Surface characteristics of the workpiece after EDM (**a**) for Experiment 20 (**b**) for Experiment 5.

**Figure 10:**
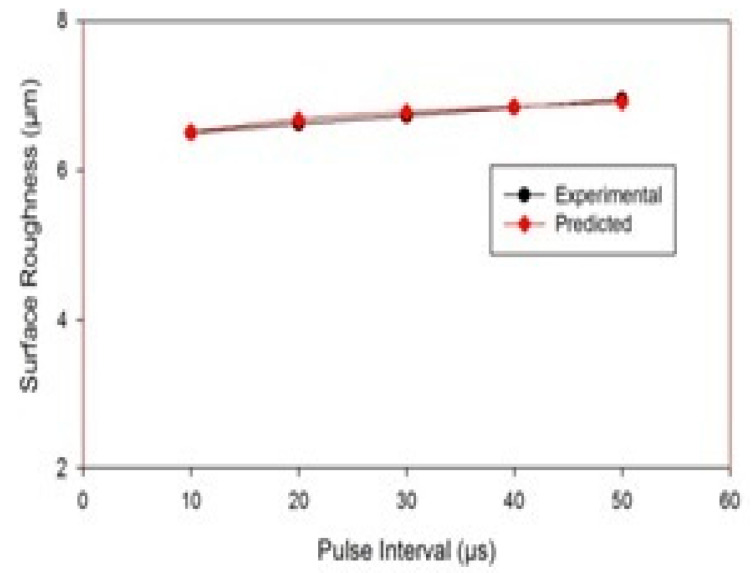
Variation in surface roughness with a pulse interval.

Figure 9 represents the influence of pulse interval on surface roughness. The SEM images show that the surface irregularities increased with the increase in pulse-off duration. During shorter pulse-off times, the flushing process is affected because, even before the debris has been cleared, fresh machining starts. This results in the resolidification of the debris. However, with the larger pulse-off time, there is a substantial temperature drop on the tool and work surface [39,40,41,42]. Therefore, the energy requirement to restore plasma channel increases. This reduces the available net energy and increases the surface irregularities. Figure 10 also indicates that an increment in pulse interval tends to a rise in the surface roughness, and the experimental and predicted surface roughness have good agreement.

## 5. Conclusions

A sintered cermet tooltip with 75% copper and 25% titanium carbide was created and used as a tool electrode for cutting H13 steel in this study. In addition, by experiment trials, the influence of machining variables was studied on the machined surface roughness, and a model has been presented, employing dimensional analysis. The following main inferences are drawn.

- Pellets were fabricated successfully from the powder metallurgy route, using copper and titanium carbide powders. SEM images signify that Cu particles had dendritic shapes, while TiC particles had sharp edges. The pellets had acceptable hardness and electrical as well as the thermal conductivity. A brazing operation was performed to braze the copper rod with the Cu–TiC tool tip, which was further used as an EDM electrode.
- Dimensional analysis was used to develop the model for predicting surface roughness by recognizing the physical, electrical, and thermal parameters that influence the surface roughness in the EDM process.
- Buckingham’s π theorem has been utilized for developing a predictive semi-empirical model of surface roughness. The developed model for surface roughness prediction was confirmed, and it showed good agreement with experimental findings.
- The roughness of the work surface tends to increase with an increase in pulse current, pulse duration, and pulse interval.

## Figures and Tables

**Table 1:** H13 steel composition (wt.%).

Carbon	Silicon	Chromium	Vanadium	Manganese	Molybdenum	Iron
1.47	0.83	4.92	0.85	0.51	1.16	Rest

**Table 2:** Measured properties (average values) of Cu–TiC.

Physical Quantity	Symbol	Units	Tool Material Properties (Cu–TiC)
Electrical Resistivity	R	Ωm	9.45 × 10^−8^
Density	P	Kg/m^3^	7439.60 ± 28.02
Thermal Conductivity	K	W/Mk	75.96 ± 1.14
Hardness	HV	HV	106.78 ± 3.67

**Table 3:** Ranges of process parameters.

Factors	Units	Range
I_p_	A	3, 5, 7, 9, 11
T_on_	µs	100, 200, 300, 400, 500
T_off_	µs	10, 20, 30, 40, 50

**Table 4:** Experimental response of surface roughness.

Exp. No.	Pulse Current (A)	Pulse Duration (µs)	Pulse Interval (µs)	Surface Roughness (µm)
1	7	300	30	7.65
2	7	500	30	8.11
3	7	300	30	7.59
4	9	200	40	8.26
5	7	300	50	7.93
6	9	400	40	8.69
7	9	400	20	8.43
8	7	300	30	7.63
9	5	400	40	6.89
10	3	300	30	5.58
11	7	100	30	7.33
12	9	200	20	7.95
13	5	200	20	6.64
14	7	300	30	7.61
15	5	200	40	6.75
16	5	400	20	6.82
17	7	300	30	7.60
18	7	300	30	7.62
19	11	300	30	9.14
20	7	300	10	7.41

**Table 5:** Physical properties of various characteristics.

Characteristic	Factor	Symbol	Dimensions	Value	Units
Quality	Surface Roughness	SR	L	--	μm
Parameter	Pulse current	$I_{p}$	$T^{-1}Q$	--	A
	Pulse duration	$T_{\mathrm{on}}$	T	--	μs
	Pulse interval	$T_{\mathrm{off}}$	T	--	μs
Material	Electrical resistivity	R	${\mathrm{ML}}^{3}T^{-1}Q^{-2}$	9.45 × 10^−8^	Ωm
	Thermal Conductivity	κ	${\mathrm{MLT}}^{-3}\theta^{-1}$	75.96	W/mk
	The density of the material	ρ	${\mathrm{ML}}^{-3}$	7439.6	$\mathrm{kg}/m^{3}$
	Hardness	HV	${\mathrm{ML}}^{-1}T^{-2}$	106.78	MPa
	Linear thermal expansion coefficient	∝	$\theta^{-1}$	13.41 × 10^−6^	1/k

**Table 6:** Parameter dimensions.

Dimension	INDEX								
	K1 (SR)	$K2\;\left(I_{p}\right)$	$K3\;\left(T_{\mathrm{on}}\right)$	$K4\;\left(T_{\mathrm{off}}\right)$	K5 (R)	K6 (κ)	K7 (ρ)	K8 (HV)	K9 (α)
M	0	0	0	0	1	1	1	1	0
L	1	0	0	0	3	1	−3	−1	0
T	0	−1	1	1	−1	−3	0	−2	0
θ	0	0	0	0	0	−1	0	0	−1
Q	0	1	0	0	−2	0	0	0	0

**Table 7:** Dimensional analysis results.

Solutions	K1 (SR)	$K2\;\left(I_{p}\right)$	$K3\;\left(T_{\mathrm{on}}\right)$	$K4\;\left(T_{\mathrm{off}}\right)$	K5 (R)	K6 (κ)	K7 (ρ)	K8 (HV)	K9 (α)
$\pi_{1}$	1	0	0	0	0	−1	−1/2	3/2	1
$\pi_{2}$	0	1	0	0	½	−3/2	−1/2	3/2	3/2
$\pi_{3}$	0	0	1	0	0	−1	−1	2	1
$\pi_{4}$	0	0	0	1	0	−1	−1	2	1

**Table 8:** Surface roughness model validation.

Pulse Current	Pulse Duration	Pulse Interval	Experimental Surface Roughness (µm)	Predicted Surface Roughness (µm)	Error (%)
6 A	250 μs	25 μs	7.04	7.13	1.28
10 A	450 μs	45 μs	8.89	9.07	2.02

## Data Availability

Relevant data are available with corresponding author.

## References

[1] Walia A.S., Srivastava V., Jain V. (2017). Fabrication and Application of Composite Electrodes in Electrical Discharge Machining—A Review. Int. J. Comput. Appl..

[2] Ong P., Chong C.H., Bin Rahim M.Z., Lee W.K., Sia C.K., Bin Ahmad M.A.H. (2018). Intelligent approach for process modelling and optimization on electrical discharge machining of polycrystalline diamond. J. Intell. Manuf..

[3] Chandrashekarappa M.P.G., Kumar S., Jagadish J., Pimenov D., Giasin K. (2021). Experimental Analysis and Optimization of EDM Parameters on HcHcr Steel in Context with Different Electrodes and Dielectric Fluids Using Hybrid Taguchi-Based PCA-Utility and CRITIC-Utility Approaches. Metals.

[4] Sen B., Hussain S.A.I., Das Gupta A., Gupta M.K., Pimenov D.Y., Mikołajczyk T. (2020). Application of Type-2 Fuzzy AHP-ARAS for Selecting Optimal WEDM Parameters. Metals.

[5] Somani N., Kumar K., Gupta N. (2020). Review on Microwave Cladding: A New Approach. Recent Advances in Computational Mechanics and Simulations.

[6] Somani N., Singh N., Gupta N.K. (2021). Joining and characterization of SS-430 using microwave hybrid heating technique. J. Eng. Des. Technol..

[7] Walia A.S., Srivastava V., Jain V., Bansal S.A. (2020). Effect of TiC Reinforcement in the Copper Tool on Roundness during EDM Process. Recent Advances in Computational Mechanics and Simulations.

[8] Walia A.S., Jain V., Srivastava V. (2019). Development and performance evaluation of sintered tool tip while EDMing of hardened steel. Mater. Res. Express.

[9] Somani N., Tyagi Y.K., Kumar P. (2021). Review on alternative approaches to fabricate the Copper based Electric Discharge Machining (EDM) electrodes. Proceedings of the IOP Conference Series: Materials Science and Engineering.

[10] Fenggou C., Dayong Y. (2004). The study of high efficiency and intelligent optimization system in EDM sinking process. J. Mater. Process. Technol..

[11] Mandal D., Pal S.K., Saha P. (2007). Modeling of electrical discharge machining process using back propagation neural network and multi-objective optimization using non-dominating sorting genetic algorithm-II. J. Mater. Process. Technol..

[12] Khan A.R., Rahman M., Kadirgama K. (2014). Neural Network Modeling and Analysis for Surface Characteristics in Electrical Discharge Machining. Procedia Eng..

[13] Payal H., Maheshwari S., Bharti P.S. (2017). Process modeling of electric discharge machining of Inconel 825 using artificial neural network. Int. J. Mech. Aerosp. Ind. Mechatron. Manuf. Eng..

[14] Guu Y.H., Chou C.Y., Chiou S.-T. (2005). Study of the Effect of Machining Parameters on the Machining Characteristics in Electrical Discharge Machining of Fe-Mn-Al Alloy. Mater. Manuf. Process..

[15] Raja S.B., Pramod C.V.S., Krishna K.V., Ragunathan A., Vinesh S. (2015). Optimization of electrical discharge machining parameters on hardened die steel using Firefly Algorithm. Eng. Comput..

[16] Pérez C.J.L. (2020). Using a Fuzzy Inference System to Obtain Technological Tables for Electrical Discharge Machining Processes. Mathematics.

[17] Mahalingam S., Kuppusamy B., Natarajan Y. (2021). Multi-objective Soft Computing Approaches to Evaluate the Performance of Abrasive Water Jet Drilling Parameters on Die Steel. Arab. J. Sci. Eng..

[18] Sahu S.K., Naik S., Das S.R., Dhupal D. (2019). Parametric Optimization of Surface Roughness and Overcut in Electric Discharge Machining of Al-SiC Using Copper Electrode. Renewable Energy and Its Innovative Technologies.

[19] Marichamy S., Saravanan M., Ravichandran M., Veerappan G. (2016). Parametric optimization of electrical discharge machining process on α–β brass using grey relational analysis. J. Mater. Res..

[20] Joshi S.N., Pande S.S. (2009). Development of an intelligent process model for EDM. Int. J. Adv. Manuf. Technol..

[21] Tsai K.-M., Wang P. (2001). Semi-empirical model of surface finish on electrical discharge machining. Int. J. Mach. Tools Manuf..

[22] Somani N., Gupta N.K. (2021). Effect of TiC nanoparticles on microstructural and tribological properties of Cu-TiC nano-composites. Proc. Inst. Mech. Eng. Part B J. Eng. Manuf..

[23] Patil N.G., Brahmankar P.K. (2010). Determination of material removal rate in wire electro-discharge machining of metal matrix composites using dimensional analysis. Int. J. Adv. Manuf. Technol..

[24] Markopoulos A., Manolakos D.E., Vaxevanidis N.M. (2008). Artificial neural network models for the prediction of surface roughness in electrical discharge machining. J. Intell. Manuf..

[25] Bobbili R., Madhu V., Gogia A. (2015). Modelling and analysis of material removal rate and surface roughness in wire-cut EDM of armour materials. Eng. Sci. Technol. Int. J..

[26] Talla G., Sahoo D.K., Gangopadhyay S., Biswas C. (2015). Modeling and multi-objective optimization of powder mixed electric discharge machining process of aluminum/alumina metal matrix composite. Eng. Sci. Technol. Int. J..

[27] Guo Y., Wang L., Zhang G., Hou P. (2016). Multi-response optimization of the electrical discharge machining of insulating zirconia. Mater. Manuf. Process..

[28] Mohanty C.P., Mahapatra S.S., Singh M.R. (2017). An intelligent approach to optimize the EDM process parameters using utility concept and QPSO algorithm. Eng. Sci. Technol. Int. J..

[29] Janmanee P., Kumjing S. (2017). A study of tungsten carbide surfaces during the electrical discharge machining using artificial neural network model. Int. J. Appl. Eng. Res..

[30] Walia A.S., Srivastava V., Jain V., Garg M. (2020). Modelling and Analysis of Change in Shape of sintered Cu–TiC tool tip during Electrical Discharge Machining process. Smart Technologies for Energy, Environment and Sustainable Development.

[31] Belloufi A., Mezoudj M., Abdelkrim M., Rezgui I., Chiba E. (2020). Experimental and predictive study by multi-output fuzzy model of electrical discharge machining performances. Int. J. Adv. Manuf. Technol..

[32] Zaw H., Fuh J., Nee A., Lu L. (1999). Formation of a new EDM electrode material using sintering techniques. J. Mater. Process. Technol..

[33] Jeswani M. (1979). Dimensional analysis of tool wear in electrical discharge machining. Wear.

[34] Lytvynenko I., Maruschak P.O., Lupenko S. (2014). Processing and modeling of ordered relief at the surface of heat-resistant steels after laser irradiation as a cyclic random process. Autom. Control. Comput. Sci..

[35] Wu K.L., Yan B.H., Huang F.Y., Chen S.C. (2005). Improvement of surface finish on SKD steel using electro-discharge machining with aluminum and surfactant added dielectric. Int. J. Mach. Tools Manuf..

[36] Buckingham E. (1914). On Physically Similar Systems; Illustrations of the Use of Dimensional Equations. Phys. Rev..

[37] Langhaar H.L., Robert E. (1980). Dimensional Analysis and Theory of Models.

[38] Khan A.R., Rahman M., Kadirgama K. (2014). An experimental investigation on surface finish in die-sinking EDM of Ti-5Al-2.5Sn. Int. J. Adv. Manuf. Technol..

[39] Walia A.S., Srivastava V., Jain V. (2019). Impact of copper-titanium carbide tooltip on machined surface integrity during electrical discharge machining of EN31 steel. Mater. Res. Express.

[40] Pandey P., Jilani S. (1986). Plasma channel growth and the resolidified layer in edm. Precis. Eng..

[41] Van Dijck F.S., Dutré W.L. (1974). Heat conduction model for the calculation of the volume of molten metal in electric discharges. J. Phys. D Appl. Phys..

[42] Khan M.A.R., Rahman M.M. (2017). Surface characteristics of Ti-5Al-2.5 Sn in electrical discharge machining using negative polarity of electrode. Int. J. Adv. Manuf. Tech..

